# Frailty incidence by diabetes treatment regimens in older adults with diabetes mellitus in the ASPirin in Reducing Events in the Elderly Study

**DOI:** 10.1007/s11357-025-01598-6

**Published:** 2025-03-17

**Authors:** Sara E. Espinoza, Jonathan C. Broder, Rory Wolfe, Michael E. Ernst, Raj C. Shah, Suzanne G. Orchard, Robyn L. Woods, Joanne Ryan, Anne Murray

**Affiliations:** 1 Center for Translational Geroscience, Diabetes and Aging Center, Department of Medicine, Cedars-Sinai Medical Center, 8700 Beverly Boulevard, Suite B113, Los Angeles, CA 90048 USA; 2 School of Public Health and Preventive Medicine, Monash University, 553 St Kilda Road, Melbourne, VIC 3004 Australia; 3 Department of Pharmacy Practice and Science, College of Pharmacy, University of Iowa, Iowa City, IA USA; 4 Department of Family Medicine, Carver College of Medicine, University of Iowa, Iowa City, IA USA; 5 Department of Family and Preventive Medicine, Rush University, Chicago, IL USA; 6 Rush Alzheimer’s Disease Center, Rush University, Chicago, IL USA; 7 Berman Center for Outcomes & Clinical Research, Hennepin Healthcare Research Institute, Minneapolis, MN USA; 8 Department of Medicine, Geriatrics Division, Hennepin Healthcare, Minneapolis, MN USA

**Keywords:** Metformin, Diabetes, Frailty, Older adults

## Abstract

Diabetes mellitus is a major risk factor for frailty in older adults, and studies suggest that frailty risk may differ by diabetes treatment regimen. To investigate the association between diabetes medication use and frailty, we conducted an observational cohort analysis of older adults with diabetes enrolled in the ASPirin in Reducing Events in the Elderly (ASPREE) study. Diabetes at baseline (*N* = 2045) was defined as self-reported diabetes, fasting blood glucose levels > 125 mg/dL, or use of diabetes medication. Diabetes medication exposure at baseline was categorized as use of metformin only (monotherapy) (*N* = 545), metformin combined with other diabetes medications (*N* = 420), other diabetes medications only (*N* = 200), or no diabetes medications (*N* = 880). Frailty was defined using a modified Fried frailty phenotype (presence of ≥ 3 of 5 criteria) and a deficit accumulation frailty index (FI, score > 0.21/1.00). Mixed effects ordinal logistic regression models revealed the odds of frailty at baseline were highest for the other diabetes medications only group, but this difference remained consistent over follow-up. After adjustment for covariates, including baseline pre-frailty, no differences in the rates of Fried or FI frailty were observed among the diabetes medication exposure groups. These findings suggest that diabetes medication exposure in older adults with diabetes does not directly impact frailty risk.

**Graphical abstract:**

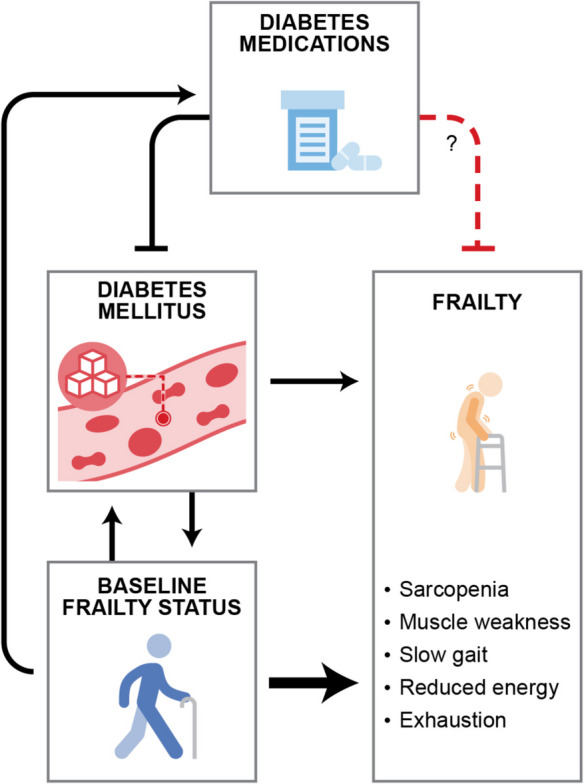

**Supplementary Information:**

The online version contains supplementary material available at 10.1007/s11357-025-01598-6.

## Background

Diabetes mellitus [1–3], an age-related disease that afflicts over one third of older adults, [4] is one of the most significant risk factors for frailty, a geriatric syndrome that poses a significant public health concern and a threat to healthy aging and independent living [5]. Frailty in older adults has been described as a syndrome of increased vulnerability to stressors and physical decline, increasing risk for disability, nursing home placement, and death [6]. Indeed, older adults with diabetes are at more than twice the risk of worsening of frailty compared to those without diabetes, which leads to increased adverse health outcomes and increased healthcare utilization [3, 7–9]. Given the high prevalence in older adults, diabetes mellitus is one of the most significant risk factors for frailty, and there is increasing interest in the potential role of diabetes treatments to enhance independence and quality of life with aging [10].

Presently, the American Diabetes Association recommends individualized diabetes treatment decisions for older adults with diabetes, taking patients’ existing comorbid conditions and functional status into account [4, 11]. However, there are limited data to specifically inform treatment decisions, especially with regard to how best to reduce geriatric syndromes such as frailty. Observational, longitudinal studies of older adults may shed light on this question, and prior observational studies have suggested that diabetes treatment regimens have differing effects on health outcomes with aging, with some evidence suggesting that metformin may reduce frailty in this population [12–15]. With the emergence of the translational geroscience field, there has been increasing interest in metformin as well as other diabetes treatments as agents that may be repurposed to improve health with aging and extend healthy years of life, or healthspan, in older adults, because of their effects on several cellular and molecular mechanisms underlying aging as well as healthspan in preclinical studies [16–20]. However, early phase clinical trials are underway to directly test these hypotheses [21, 22].

In this study, we sought to examine the association between diabetes treatment regimens and frailty incidence in participants with diabetes mellitus enrolled in the ASPirin in Reducing Events in the Elderly (ASPREE) clinical trial.

## Methods

### Study population: the ASPREE trial

ASPREE was a double-blind, placebo-controlled trial of low-dose aspirin (100 mg) vs. placebo for the prevention of dementia-free and physical disability-free survival in a population of relatively healthy community-dwelling older adults. Details about the recruitment of participants, main results, and participant baseline characteristics have been previously described [23–25]. ASPREE enrolled adults aged 70 years or older (65 years or older for US ethnic minorities), in the USA and Australia, without overt cardiovascular disease, dementia, and independence-limiting physical disability, and expected survival for at least 5 years. Participants were followed in-person annually to conduct assessment for all measures included in this analysis, as previously described [23]. Telephone follow-up was also conducted every 6 months between in-person visits. The study was approved by the Institutional Review Boards at each study site, and all participants provided written informed consent prior to enrollment.

### Baseline measurements

Demographic information, including age, sex, race, ethnic group, and medical history, were collected during the baseline (i.e., enrollment) in-person study visits. Lifestyle factors were also assessed, including smoking and alcohol usage (current and past). Blood pressure (average of three measurements), height, waist circumference, and weight were directly measured and recorded. A fasting clinical chemistry panel was collected and included glucose, lipids (low density lipoprotein, high density lipoprotein, triglycerides), hemoglobin, and creatinine, and a urine sample for albumin-creatinine ratio. Chronic kidney disease (CKD) was defined in ASPREE as urine albumin creatinine ratio of 3 mg/mmol or more, or estimated glomerular filtration rate (eGFR) of less than 60 mL per minute per 1.73 m^2^. eGFR was calculated using serum creatinine assessed from clinical laboratory measures as previously described [26]. Global cognition was measured using the Modified Mini-Mental State examination (3MS) [27]. Diabetes mellitus (*n* = 2045) was defined in ASPREE as self-reported diabetes, elevated fasting blood glucose levels (> 125 mg/dL), or use of a diabetes medication at baseline as described [28].

### Frailty assessment

#### Modified Fried frailty phenotype

A modified Fried frailty phenotype was defined at baseline and each annual visit (from baseline to a maximum of seven annual visits) based on the following criteria: slow gait speed based on 3-m walk; weak grip strength assessed by dynamometer; low body mass index (< 20 kg/m^2^); self-reported feelings of exhaustion based on a question from the Center for Epidemiologic Studies Depression (CES-D 10) 10-item scale; and low physical activity (either from no reported walking outside home, or less than 10 min walking outside home without sitting down), as previously described [29]. Participants with three or more of these conditions were considered ‘Frail’, while participants with one or two conditions were considered ‘Pre-frail’. Participants needed to complete all five components to be defined into the three frailty categories (not frail, pre-frail, or frail). Due to the ASPREE data collection schedule, some components of frailty were not collected at every annual visit. Measures of physical performance (gait speed and grip strength) were collected at baseline and then at years 2, 4, and 6, and CES-D 10 was collected at baseline and then at years 1, 3, 4, 5, 6, and 7; all measures were collected at a milestone visit (2017) that was year 3, 4, 5, 6, or 7 depending on the year of each participant’s study enrolment. Thus, missing data were mostly missing by design and was imputed using time series imputation via the R package ImputeTS [30], as previously described [29, 31]. A Fried frailty endpoint occurred when the participant first reached criteria for frailty (i.e., the presence of three or more of the frailty characteristics) at any follow-up annual visit. Participants were censored at the last annual visit where they had a Fried frailty score.

#### Deficit accumulation Frailty Index (FI)

Deficit accumulation Frailty Index (FI) at baseline and each annual visit was defined according to up to 67 deficits regarding general health, chronic conditions, physical limitations, and cognitive deficits, as previously described [31]. Briefly, the items used data collected at baseline across multiple domains, including sociodemographic factors, lifestyle factors, chronic medical conditions, morbidities, physical activity, functional engagement, mental health, cognition, laboratory/pathology values, and self-rated health status. For each of the items, a complete deficit was coded as 1 and an absence was coded as 0. A number of items were also not binary and had a degree of deficit, which were coded with an intermediate score between 0 and 1. The overall frailty index at an annual visit was determined by the mean score (from 0 to 1) of the items completed (maximum of 67), which were weighted equally. Participants had to complete at least 50 items; otherwise, their FI score was considered missing. To be categorized as frail, participants had to have a frailty index score of greater than 0.21 (out of 1), while pre-frail was considered for participants who had a frailty index score greater than 0.1 and less than and equal to 0.21 [32]. Grip strength and gait speed as components of the frailty index were also imputed using the same process that was applied to Fried frailty phenotype. A FI-defined frailty endpoint occurred when the participant first reached a FI score of greater than 0.21. Participants were censored at the last annual visit where they had a FI score.

### Diabetes medication exposure

Medication usage was ascertained at ASPREE enrollment by participant interview and review of medication bottles brought to the baseline study visit. Study staff also reviewed prescribed medications through review of the medical record whenever possible. Medications were reviewed and updated at all study follow-up visits. These medications were then classified according to the World Health Organization Anatomical Therapeutic Chemical system (WHO-ATC).

We classified diabetes medication as informed by practice guidelines at the time of the ASPREE enrollment period, which recommended lifestyle modification alone as first-line, followed by the addition of metformin as second-line, and the addition of a second diabetes medication as third-line [33]. Thus, diabetes medication exposure was categorized into four categories: no diabetes medications, metformin only, combined metformin and other diabetes medication, and other diabetes medications only. ‘Metformin only’ refers to the prescription at baseline of a medication with an ATC code of A10BA02, and no other diabetes medications. ‘Other diabetes medication monotherapy’ refers to use of insulin (A10A), sulfonylureas (A10BB), alpha glucosidase inhibitors (A10BF), thiazolidinediones (A10BG), DDP-4 inhibitors (A10BH), GLP-1 analogues (A10BJ), SGLT2 inhibitors (A10BK), or other (A10BX) at baseline. ‘Combined Metformin and other diabetes medications’ includes participants who used both metformin and other diabetes medications at baseline. Only *baseline* diabetes medication exposure was considered because 75% of participants with diabetes remained within their diabetes medication exposure group from baseline to the end of follow-up.

### Statistical analysis

Participant characteristics were calculated by diabetes medication exposure group. Kaplan–Meier cumulative incidence curves were presented by diabetes exposure group for imputed Fried frailty and FI frailty endpoints. Cox proportional hazards regression was performed to assess time to imputed Fried frailty (three or more Fried phenotype criteria) and imputed FI-defined frailty (frailty index score of greater than 0.21) endpoints, with ‘other diabetes medications only’ as the reference for comparisons among diabetes medication exposure groups. This reference ensured that metformin groups were compared with other individuals also taking diabetes medications as the primary comparisons of interest. If a participant was frail at baseline or had missing frailty data at baseline, they were excluded from the Cox proportional hazards regression models and cumulative incidence plots on the frailty endpoints. The Cox proportional hazards regression models were adjusted for baseline variables, including age, sex, education, ethnicity, alcohol use, antihypertensive use, SBP, DBP, triglycerides, HDL, LDL, statin use, hemoglobin, eGFR, polypharmacy (5 + medications), 3MS, fasting blood glucose, BMI, and baseline frailty score (Fried: 0, 1, 2; or FI: 0–0.21). In order to assess whether there is a potential reverse causation relationship between diabetic medication exposure group and frailty, a second set of analyses were conducted on both Fried and FI frailty without adjustment for baseline pre-frailty score. The proportional hazards assumption was inspected by visualizing Schoenfeld residuals across time, and these indicated that the assumption was not breached. For sensitivity analysis, the models were repeated for non-imputed frailty endpoints.

A secondary analysis was conducted to assess the change across all frailty categories (non-frail to pre-frail to frail) over time/annual visits, using mixed effects ordinal logistic regression on imputed Fried frailty phenotype and FI frailty categories across annual visits. Participants with an incident frailty endpoint or who had missing frailty data at baseline were included in these models. Ordinal logistic regression estimates the cumulative log odds of the frailty categories (frail > pre-frail > non-frail). The odds ratios from the predictors (e.g., diabetes medication exposure) are for frail or pre-frail vs not frail (odds ≥ pre-frail), or frail vs pre-frail or not frail (odds ≥ frail). The model assumes these two odds ratios are the same (proportional odds assumption) [34]. Interaction effects between diabetes exposure and time/years/annual visit (0 [baseline], 1, 3, 4, 5, 6 years) were also included to assess whether the odds of being in higher frailty categories by metformin exposure changed over time. The mixed effects ordinal logistic regression models were adjusted for baseline age, sex, education (≥ 12 years, < 12 years), ethnicity, alcohol use (current, former, never), antihypertensive use, SBP, DBP, triglycerides, HDL, LDL, statins use (yes, no), hemoglobin, eGFR, polypharmacy (5 + medications) (yes, no), 3MS, fasting blood glucose, and BMI. Predicted probabilities of imputed Fried frailty and FI-defined frailty categories over time by metformin exposure were conducted and visualized from the mixed effects ordinal logistic regression models. The proportional odds assumption was assessed by inspecting the cut-point specific odd ratios (OR) (frail vs pre-frail + not frail, and frail + pre-frail vs not frail), which indicated that the estimates were largely consistent, and the proportional odds assumption was not breached. All analyses were conducted using R [30].

## Results

Of the 19,114 participants randomized in ASPREE, 2045 individuals with diabetes were included in this study (Supplemental Fig. 1). Of these participants, 43.0% were not using any diabetes medications (‘no diabetes medications’), 26.7% used ‘metformin only,’ 20.5% used ‘metformin combined with other diabetes medications,’ and 9.8% used ‘other diabetes medications only.’ The most commonly used other diabetes medication classes were insulin, then sulfonylureas (gliclazide), followed by sitagliptin, or a dipeptidyl peptidase-4 inhibitor (Supplemental Table 1). Participants on ‘metformin combined with other diabetes medications’ were most likely to use gliclazide, followed by insulin and sitagliptin and had lower HDL and were more likely to have hypertension and polypharmacy and to use statins, compared to the other groups. Individuals using ‘diabetes medications other than metformin’ were less likely to be white and more likely to have greater years of education compared to the other groups (Table 1), and were more likely to have lower eGFR and higher fasting glucose, whereas users of ‘metformin only’ had the highest triglyceride levels. Further, individuals taking ‘other diabetes medications only’ had higher fasting glucose and were more likely to have low eGFR and frailty at baseline compared to the other groups, using both Fried and frailty index definitions. No differences in disease history of cancer, CKD, osteoarthritis, depression, or asthma were observed between the groups.

**Fig. 1:**
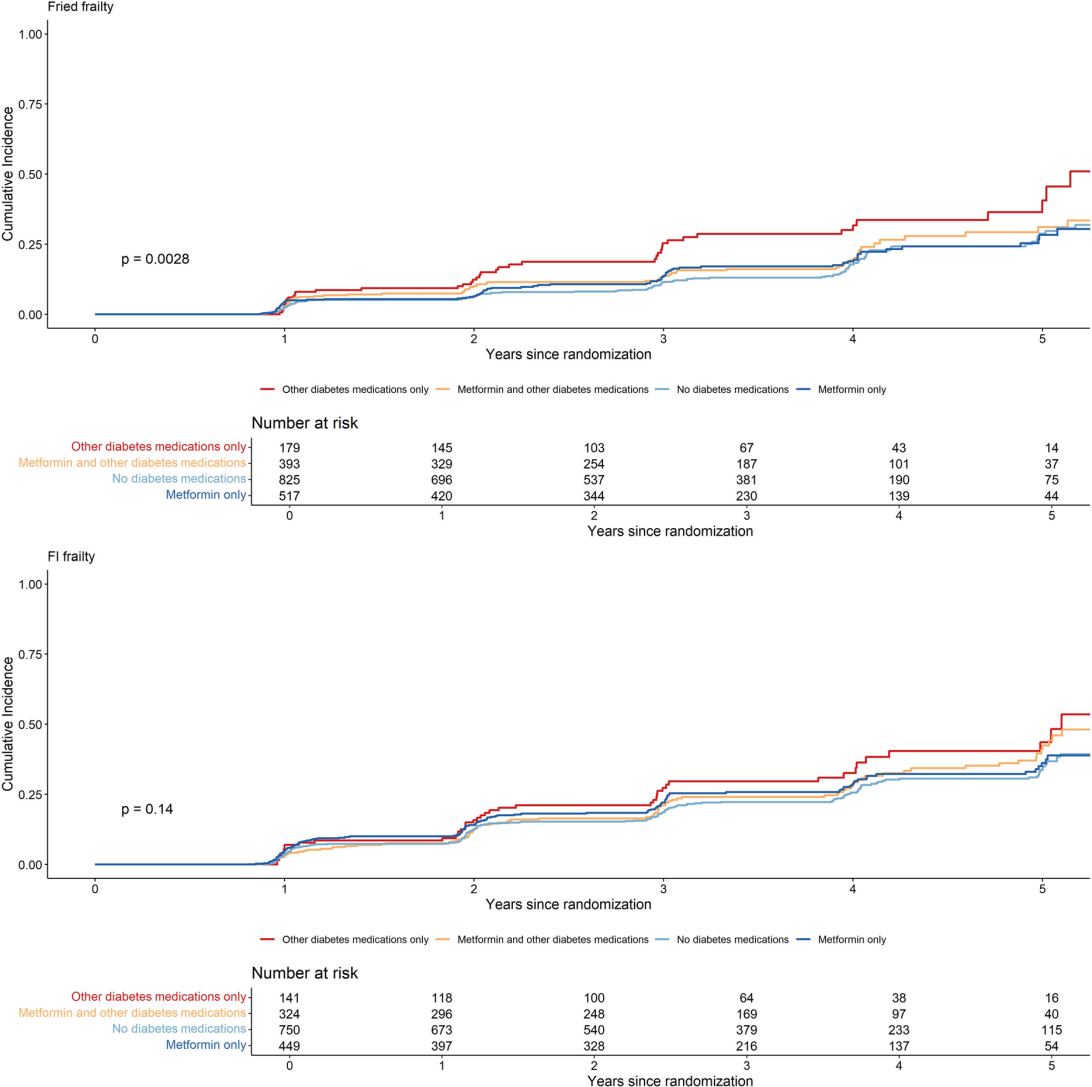
Kaplan–Meier cumulative incidence curves for imputed Fried phenotype and deficit accumulation FI-defined frail endpoints by metformin exposure group in those with diabetes. *P* values were calculated using Logrank test

**Table 1:** Characteristics of participants with diabetes at baseline by diabetes medication exposure

Characteristics	Metformin only (*N* = 545)	No diabetes medications (*N* = 880)	Metformin and other diabetes medications (*N* = 420)	Other diabetes medications only (*N* = 200)	*P* value
	Mean (SD) or *N* (%)	Mean (SD) or *N* (%)	Mean (SD) or *N* (%)	Mean (SD) or *N* (%)	
Age (years)^ab^	74.3 (4.8)	74.9 (4.8)	73.8 (4.5)	74.7 (5.4)	0.001
Male^c^	265 (49%)	440 (50%)	232 (55%)	109 (54%)	0.135
Education ≥ 12 years^c^	279 (51%)	414 (47%)	223 (53%)	118 (59%)	0.011
Ethnicity white^c^	424 (78%)	783 (89%)	331 (79%)	126 (63%)	< 0.001
BMI ≥ 25 kg/m^2c^	479 (88%)	745 (85%)	375 (89%)	172 (87%)	0.127
Smoking^c^					0.604
- Current	31 (6%)	39 (4%)	16 (4%)	12 (6%)	
- Former	246 (45%)	390 (44%)	178 (42%)	92 (46%)	
- Never	268 (49%)	451 (51%)	226 (54%)	96 (48%)	
Alcohol use^c^					< 0.001
- Current	350 (64%)	650 (74%)	263 (63%)	117 (58%)	
- Former	55 (10%)	59 (7%)	50 (12%)	24 (12%)	
- Never	140 (26%)	171 (19%)	107 (25%)	59 (30%)	
Systolic blood pressure (mmHg)^ab^	139.2 (15.8)	141.1 (16.3)	140.0 (15.7)	140.9 (17.3)	0.160
Diastolic blood pressure (mmHg)^ab^	76.8 (9.7)	76.5 (10.0)	76.4 (9.3)	74.6 (9.9)	0.048
Anti-hypertensive use^c^^e^	417 (77%)	604 (69%)	343 (82%)	165 (82%)	< 0.001
Polypharmacy (≥ 5 medications)^c^	316 (58%)	301 (34%)	325 (77%)	123 (62%)	< 0.001
Statin use^c^	361 (66%)	411 (47%)	303 (72%)	126 (63%)	< 0.001
eGFR^ab^^d^	73.7 (15.1)	70.8 (15.2)	72.0 (15.8)	67.7 (17.8)	< 0.001
LDL (mg/dL)^ab^	91.2 (32.5)	104.1 (32.8)	83.5 (31.9)	91.0 (30.5)	< 0.001
HDL (mg/dL)^ab^	52.5 (14.9)	55.8 (16.9)	49.8 (12.9)	53.7 (19.0)	< 0.001
Glucose (mg/dL)^ab^	127.0 (30.3)	126.2 (27.0)	140.4 (44.0)	144.3 (56.3)	< 0.001
Triglyceride (mg/dL)^ab^	146.2 (78.1)	140.8 (78.1)	141.0 (70.8)	124.8 (64.0)	0.008
Hemoglobin (g/dL)^ab^	13.9 (1.3)	14.4 (1.3)	13.8 (1.3)	13.8 (1.3)	< 0.001
3MS^ab^	92.2 (4.8)	92.6 (4.7)	92.2 (4.8)	91.6 (4.9)	0.027
Cancer history^c^	113 (21%)	162 (18%)	69 (16%)	42 (21%)	0.313
Chronic kidney disease history^c^	14 (3%)	31 (4%)	8 (2%)	11 (6%)	0.078
Osteoarthritis history^c^	118 (45%)	212 (48%)	98 (49%)	31 (41%)	0.611
Depression history^c^	52 (22%)	86 (23%)	42 (24%)	27 (35%)	0.109
Asthma history^c^	62 (26%)	114 (28%)	43 (25%)	18 (24%)	0.853
Baseline Fried frailty^cf^					< 0.001
- Not frail	261 (49%)	459 (54%)	191 (46%)	66 (34%)	
- Pre-frail	256 (48%)	366 (43%)	202 (49%)	113 (58%)	
- Frail	17 (3%)	26 (3%)	18 (4%)	15 (8%)	
Baseline frailty index^cf^					< 0.001
- Not frail	133 (24%)	259 (29%)	90 (21%)	38 (19%)	
- Pre-frail	316 (58%)	491 (56%)	234 (56%)	103 (52%)	
- Frail	96 (18%)	130 (15%)	95 (23%)	59 (30%)	

Summary stats are mean (SD) for numeric characteristics or *N* (%) for categorical variables

*N* missing: BMI, 7; eGFR, 44; LDL, 73; HDL, 57; Glucose, 61**;** Triglyceride, 24; Cancer history, 2; Kidney disease history, 1; Oesteoarthritis history, 1065; Depression history, 1176; Asthma history, 1155; Baseline fried frailty, 55; Baseline frailty index, 1

^a^Mean (standard deviation)

^b^*P* value calculated using ANOVA test

^c^*P* value calculated using chi-squared test

^d^CKD formula mL/min/1.73 m^2^

^e^Antihypertensive medication use (ATC C02, C03 [excluding C03D], C07, C08, or C09)

^f^Imputed frailty

The unadjusted cumulative incidence plots (Fig. 1) showed that those on ‘other diabetes medications only’ had the highest incidence of frailty (both Fried and FI frailty) over time; however, the Log Rank test was only significant for Fried frailty. In the fully adjusted Cox proportional hazards regression models (Table 2), ‘metformin only’ users had similar rates of Fried frailty (Adj HR = 0.79, 95% CI = 0.52, 1.21) and FI frailty (Adj HR = 0.87, 95% CI = 0.60, 1.28), compared to those on ‘other diabetes medications only’. Further, no significant differences were observed in rates of Fried and FI frailty between any diabetes medication exposure group comparison in the fully adjusted analysis (Supplemental Table 2). In the analysis that did not adjust for baseline pre-frailty score, we did observe higher rates of Fried frailty in ‘other diabetes medication only’ participants compared to ‘metformin only’ and ‘no diabetes medication’ participants (Table 2). Sensitivity analyses using non-imputed endpoints were largely similar, apart from wider confidence intervals likely due to fewer endpoint numbers (Supplemental Table 2).

**Table 2:** Results of time to frailty event analysis by diabetes medication usage status

Endpoint	Metformin only	No diabetes medications	Metformin combined with other diabetes medications	Other diabetes medications only
*N* = sample size of those who are not-frail or pre-frail at baseline Number of events (rates per 1000 person-years)				
Fried frail^a^	*N* = 517 75 (51.99)	*N* = 825 111 (47.08)	*N* = 393 65 (58.23)	*N* = 179 42 (90.42)
FI frail^b^	*N* = 449 117 (84.58)	*N* = 750 178 (74.38)	*N* = 324 91 (86.95)	*N* = 141 44 (103.71)
Cox proportional-hazards regression models—hazard ratio (HR), 95% confidence interval (CI)				
*Reference group: Other diabetes medications only*				
Fried frail^ac^	0.63 (0.41, 0.97)	0.69 (0.46, 1.05)	0.79 (0.51, 1.21)	Ref
FI frail^bc^	0.72 (0.49, 1.05)	0.68 (0.47, 0.99)	0.70 (0.48, 1.03)	Ref
Fried frail (adjusting for baseline frailty score)^ac^	0.79 (0.52, 1.21)	0.94 (0.61, 1.44)	0.94 (0.61, 1.45)	Ref
FI frail (adjusting for frailty score)^bc^	0.87 (0.60, 1.28)	0.75 (0.52, 1.09)	0.87 (0.59, 1.29)	Ref

^a^Imputed fried frail endpoint is defined as the presence of three or more Fried frailty phenotype criteria. Participants with an incident frailty endpoint or missing frailty data at baseline were excluded

^b^Imputed FI frail endpoint is defined as deficit accumulation FI score of greater than 0.21. Participants with an incident frailty endpoint or missing frailty data at baseline were excluded

^c^Adjusted for age, sex, education, ethnicity, alcohol use, antihypertensive use, SBP, DBP, triglycerides, HDL, LDL, statins use, hemoglobin, eGFR, polypharmacy (5 + medications), 3MS, fasting blood glucose, and BMI

**Fig. 2:**
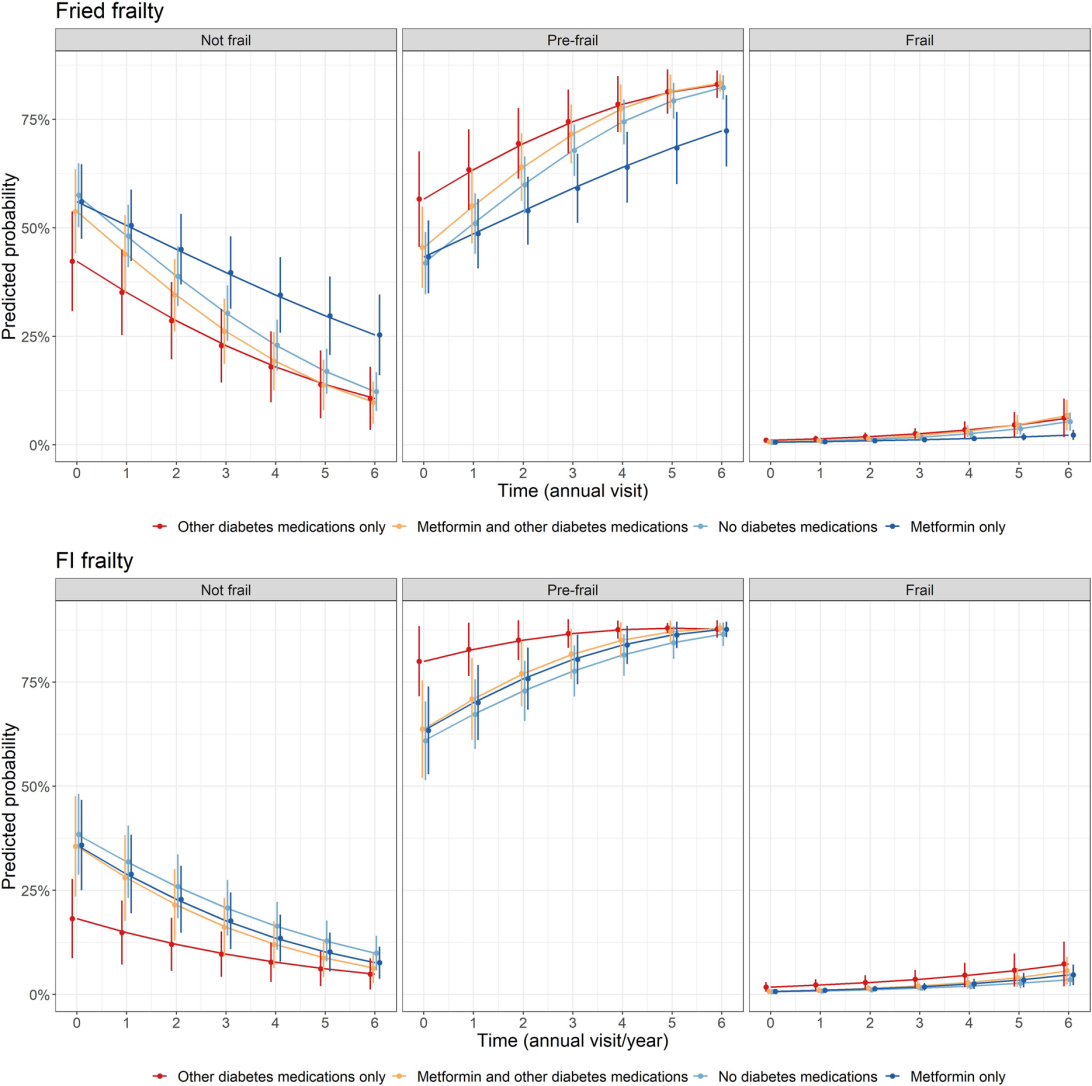
Prediction probability of frailty over time by metformin exposure in those with diabetes. Predictions are based on mixed effects ordinal logistic regression models and at mean values for numeric covariates and reference values for categorical covariates

Mixed effects ordinal logistic regression models (Table 3) showed that those on ‘metformin only’ had lower odds of worse frailty categories for both Fried frailty and frailty index frailty compared with ‘other diabetes medication only’ users at baseline. Additionally, those taking ‘metformin combined with other diabetes medications’ and those using ‘no diabetes medications’ had lower odds of being in higher Fried and frailty index frailty states (from not-frail to pre-frail/frail, or from not-frail/pre-frail to frail) at baseline, compared to those using ‘other diabetes medication only,’ and this remained consistent over the follow-up period (interactions were not significant, Fig. 2).

**Table 3:** Results of mixed effects ordinal logistic regression models for frailty category status across annual visits by diabetes medication usage in those with diabetes mellitus

	Fried frailty^ac^		FI frailty^ad^	
	OR (95% CI)^b^	*P* value^b^	OR (95% CI)^b^	*P* value^b^
Other diabetes medications only	Ref	Ref	Ref	Ref
Metformin and other diabetes medications	0.63 (0.39, 1.00)	0.050	0.40 (0.22, 0.75)	0.004
No diabetes medications	0.54 (0.35, 0.84)	0.006	0.36 (0.20, 0.64)	0.001
Metformin only	0.57 (0.37, 0.90)	0.015	0.40 (0.22, 0.72)	0.002
Year	1.35 (1.17, 1.55)	< 0.001	1.28 (1.13, 1.43)	< 0.001
Metformin and other diabetes medications*Year	1.10 (0.93, 1.30)	0.258	1.11 (0.96, 1.28)	0.149
No diabetes medications*Year	1.08 (0.93, 1.26)	0.319	1.05 (0.91, 1.20)	0.505
Metformin only*Year	0.92 (0.79, 1.08)	0.315	1.08 (0.94, 1.23)	0.293

^a^Using imputed frailty data. Participants with an incident frailty endpoint or missing frailty data at baseline were included

^b^Adjusted for age, sex, education, ethnicity, alcohol use, antihypertensive use, SBP, DBP, triglycerides, HDL, LDL, statins use, hemoglobin, CKD, polypharmacy, 3MS, fasting blood glucose, and BMI

^c^Participant specific intercepts and slopes were included in the regression models to account for individual difference in baseline and trajectories over time

^d^Due to convergence issues from missing data, only a participant specific intercept was included

**Fig. 3:**
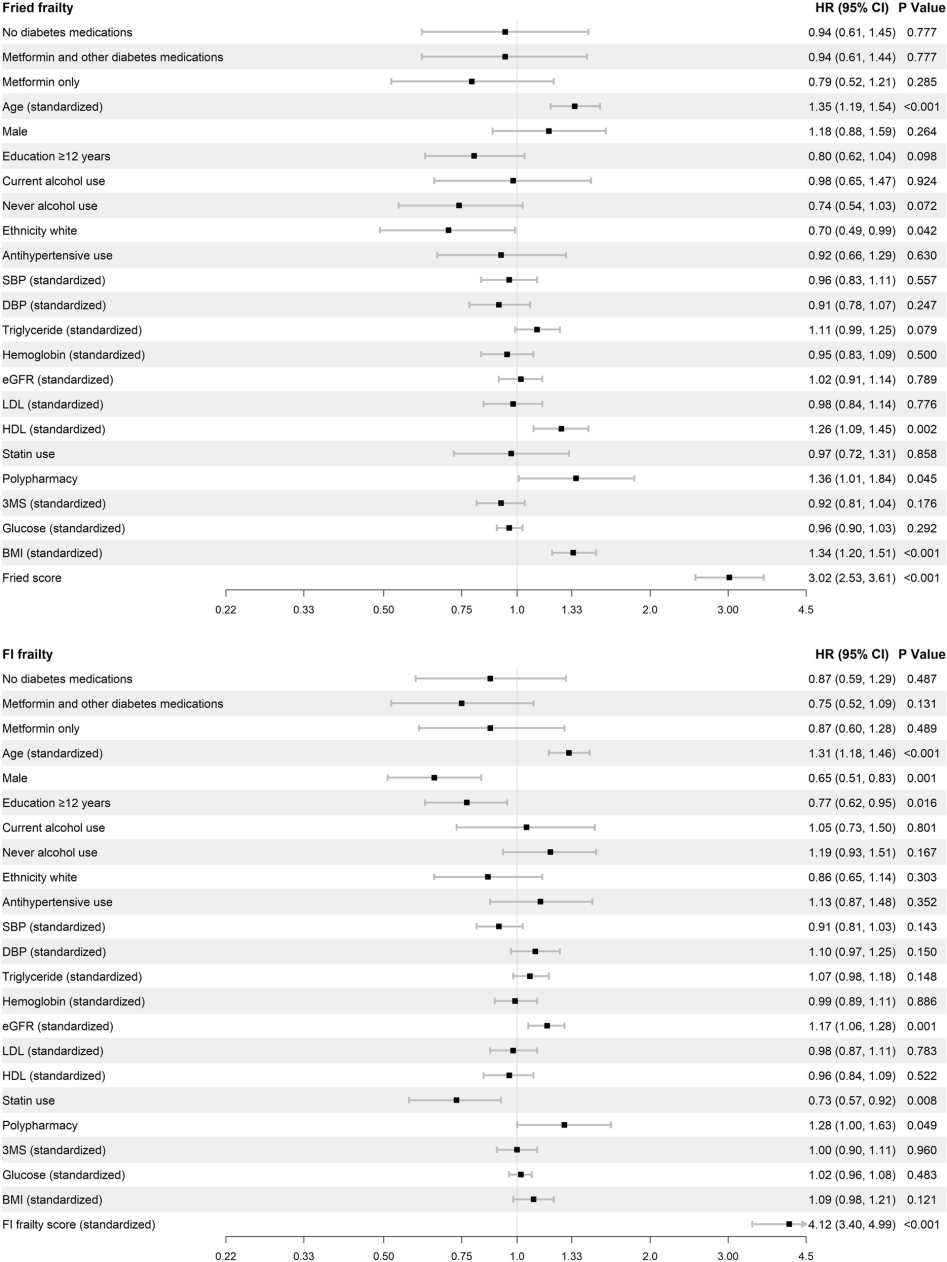
Cox proportional-hazards regression model showing coefficient estimates for all baseline covariates for the risk of frailty incidence as measured by Fried phenotype (top panel) and by frailty index (bottom panel)

From Cox proportional-hazards regression models, we found that the most significant predictors of frailty onset across both Fried phenotype and frailty index were age, polypharmacy, and baseline frailty score (Fig. 3).

## Discussion

In older adults with diabetes mellitus at baseline in ASPREE, we observed that the rates of frailty incidence (both Fried and frailty index) were similar among the different diabetes medication treatment groups after adjusting for covariates, including baseline pre-frailty which was a significant predictor of frailty incidence. We also observed no differences between diabetes medication treatment groups in the odds of higher frailty over time, though those on ‘other diabetes medications only’ had greater odds of frailty at baseline.

Insulin resistance and type 2 diabetes lead to greater declines in gait speed, muscle mass, and muscle strength in older adults [35–39], which contributes to increased risk for frailty [3, 40–43]. Evidence supports that better glycemic control in older adults with diabetes is associated with less decline in physical function, [44] and that the use of insulin sensitizing medications, such as metformin, provides potential benefit for ameliorating this decline in physical function and in lean mass in older adults with insulin resistance [45]. For example, metformin or thiazolidinedione use in older adults with diabetes or insulin resistance resulted in reduced loss of appendicular lean mass and less decline in gait speed [45, 46].

Insulin resistance is associated with an inflammatory milieu, a major hallmark of aging that is associated with frailty as well as several other age-related diseases [47–49]. Therefore, it was plausible that the use of insulin sensitizing medications such as metformin may have benefit for prevention of frailty in this high-risk group [50]. In recent years, emerging pre-clinical and observational studies have demonstrated that metformin is associated with reduced frailty, mortality, and onset of age-related diseases [14, 15, 20, 50–55], including our work in ASPREE demonstrating metformin was associated with cancer reduction [12]. Because of its known impact on several aging mechanisms, [51, 56] excellent safety profile, and because it is generally well-tolerated, metformin is now being considered a possible therapeutic agent to reduce frailty, particularly in older adults with insulin resistance [57]. However, because some studies have shown no benefit with metformin, [58–60] results are mixed, calling for clinical trials to directly test whether metformin reduces aging-focused outcomes such as frailty, cognitive dysfunction, and dementia [21, 61, 62]. With the emergence of other drug classes such as sodium-glucose cotransporter-2 (SGLT2) inhibitors and glucagon-like peptide-1 (GLP-1) agonists to treat diabetes and obesity, which also have potential to modulate aging mechanisms [18, 63], there is considerable interest in examining the impact and potential usefulness of these agents on aging hallmarks and measures of healthspan, such as frailty [19, 64].

In our fully adjusted analysis, we did not observe a benefit of metformin on Fried or frailty index frailty outcomes, or any difference between the diabetes medication exposure groups on future frailty risk. We did observe higher rates of Fried frailty in those on ‘other diabetes medication only’ when baseline Fried score was not adjusted for. Those on ‘other diabetes medications only’ were already closer to becoming frail at baseline, which may be due to more advanced diabetes at baseline, which is supported by our finding of higher fasting glucose at baseline. Although speculative, more advanced diabetes progression in those on ‘other diabetes medication only’ may be due to comorbidities resulting from diabetes progression brought on by the inability to prescribe metformin due to intolerance of metformin, which is typically first-line therapy. The results from the mixed effects models were congruent, as there were greater odds of being in higher frailty levels at baseline for those on ‘other diabetes medications only’ but this elevated risk at baseline did not further increase longitudinally. Consistent with prior studies [65], we found that baseline pre-frailty, both Fried phenotype and frailty index score, were major predictors of future frailty. Indeed, frailty is emerging as an important predictor of adverse outcomes, such as hospitalization, disability, and mortality, in older adults with diabetes [6, 65, 66], leading to consensus statements that frailty, as well as multimorbidity, should be considered when determining individualized diabetes treatment goals [67, 68].

Our study has some limitations. Firstly, we did not collect data on duration of diabetes, or medication history prior to consent for the trial. This could bias our results, potentially overestimating the rates of frailty in the ‘other diabetes medications only’ and ‘metformin combined with other diabetes medications’ groups, as these groups are likely to have more advanced diabetes. Additionally, because hemoglobin A1c was not measured in the ASPREE clinical trial, we were unable to adjust for hyperglycemia using this standard metric, instead relying on fasting blood glucose. We acknowledge that this lack of precision in assessing glycemic control may further bias our findings, potentially further exaggerating the frailty rates in the ‘other diabetes medications only’ and ‘metformin combined with other diabetes medications’ groups, as they are expected to differ in glycemia, which was confirmed by the comparison of baseline fasting glucose by groups. It is also important to note that the ‘no diabetes medications’ group may represent individuals who have undiagnosed diabetes or early diabetes. We observed that the ‘metformin only’ group was similar to those in the ‘no diabetes medications’ on fasting glucose; therefore, it is possible that our finding that risk of frailty in the ‘metformin only’ group did not differ from the ‘no diabetes medication’ group is related to less severe insulin resistance and hyperglycemia in the ‘no medications group,’ which is known to correlate with physical function in older adults [42]. We acknowledge also that we did not ascertain which type of diabetes participants had; however, because this study is of older adults [23], the prevailing diabetes type is likely to be type 2 diabetes. Furthermore, because this trial was initiated in 2010, the use of newer anti-diabetes agents, such as SGLT2 inhibitors and GLP1 agonists, was not yet common. Lastly, this is a clinical trial cohort that examined the effect of aspirin on physical disability-free and dementia-free survival; however, we previously reported that aspirin use had no effect on frailty onset in ASPREE [29].

The present study has several strengths, including a large well-characterized cohort of initially healthy community-dwelling older adults. Further, numerous demographic and risk factors relative to frailty were accounted for in the analyses, including baseline pre-frailty. Medication collection data quality was high, as participants brought in their medications at the baseline visit, which were also often reviewed using medical records. We were able to analyze both the Fried frailty phenotype and the frailty index, which provides a more nuanced account on the relationship between metformin exposure and frailty.

In summary, adjusted Cox proportional hazards models showed no significant differences in the rates of Fried frailty or the frailty index across the diabetes medication exposure groups. Similarly, mixed-effects ordinal logistic regression revealed no differences in the odds of higher frailty over time, though those on ‘other diabetes medications only’ had greater odds of frailty at baseline. Based on these findings, we conclude that diabetes medication exposure, and specifically metformin, is unlikely to impact future frailty risk. Given the high prevalence of diabetes in older adults and the impact of frailty in this population, clinicians should strongly consider the incorporation of frailty screening into routine clinical practice and identifying frailty risk factors in older adults with diabetes mellitus. [66] Further studies, including clinical trials, are needed to thoroughly examine the impact of diabetes medications on aging-focused outcomes such as frailty in older adults with diabetes.

## Supplementary Information

Below is the link to the electronic supplementary material.

Supplementary file1 (DOCX 25.4 KB)

Supplementary file2 (DOCX 128 KB)

## References

[1] Hanlon P, Fauré I, Corcoran N, et al. Frailty measurement, prevalence, incidence, and clinical implications in people with diabetes: a systematic review and study-level meta-analysis. Lancet Healthy Longev. 2020;1(3):e106–16. 33313578 10.1016/S2666-7568(20)30014-3 PMC7721684

[2] Yuan L, Chang M, Wang J. Abdominal obesity, body mass index and the risk of frailty in community-dwelling older adults: a systematic review and meta-analysis. Age Ageing. 2021;50(4):1118–28. 33693472 10.1093/ageing/afab039

[3] Espinoza SE, Jung I, Hazuda H. Frailty transitions in the San Antonio longitudinal study of aging. J Am Geriatr Soc. 2012;60(4):652–60. 22316162 10.1111/j.1532-5415.2011.03882.x PMC3325321

[4] Kalyani RR, Golden SH, Cefalu WT. Diabetes and aging: unique considerations and goals of care. Diabetes Care. 2017;40(4):440–3. 10.2337/dci17-0005. 28325794 10.2337/dci17-0005 PMC5360288

[5] Won CW, Ha E, Jeong E, et al. World Health Organization Integrated Care for Older People (ICOPE) and the Integrated Care of Older Patients with Frailty in Primary Care (ICOOP_Frail) Study in Korea. Ann Geriatr Med Res. 2021;25(1):10–6. 10.4235/agmr.21.0025. 33794585 10.4235/agmr.21.0025 PMC8024169

[6] Castro-Rodríguez M, Carnicero JA, Garcia-Garcia FJ, et al. Frailty as a major factor in the increased risk of death and disability in older people with diabetes. J Am Med Dir Assoc. 2016;17(10):949–55. 27600194 10.1016/j.jamda.2016.07.013

[7] Kong L-N, Lyu Q, Yao H-Y, Yang L, Chen S-Z. The prevalence of frailty among community-dwelling older adults with diabetes: a meta-analysis. Int J Nurs Stud. 2021;119:103952. 34022743 10.1016/j.ijnurstu.2021.103952

[8] Kalyani RR, Corriere M, Ferrucci L. Age-related and disease-related muscle loss: the effect of diabetes, obesity, and other diseases. Lancet Diabetes Endocrinol. 2014;2(10):819–29. 24731660 10.1016/S2213-8587(14)70034-8 PMC4156923

[9] Chao CT, Wang J, Chien KL. Both pre-frailty and frailty increase healthcare utilization and adverse health outcomes in patients with type 2 diabetes mellitus. Cardiovasc Diabetol. 2018;17(1):130. 10.1186/s12933-018-0772-2. 30261879 10.1186/s12933-018-0772-2 PMC6158921

[10] Kulkarni AS, Aleksic S, Berger DM, Sierra F, Kuchel GA, Barzilai N. Geroscience-guided repurposing of FDA-approved drugs to target aging: a proposed process and prioritization. Aging Cell. 2022;21(4):e13596. 35343051 10.1111/acel.13596 PMC9009114

[11] American Diabetes Association. 12. Older adults: standards of medical care in diabetes—2019. Diabetes Care. 2021;44(Supplement 1):S168–79. 33298423 10.2337/dc21-S012

[12] Orchard SG, Lockery JE, Broder JC, et al. Association of metformin, aspirin, and cancer incidence with mortality risk in adults with diabetes. JNCI Cancer Spectr. 2023;7(2):pkad017. 36857596 10.1093/jncics/pkad017 PMC10042437

[13] Wang C-P, Lorenzo C, Espinoza SE. Frailty attenuates the impact of metformin on reducing mortality in older adults with type 2 diabetes. J Endocrinol Diabetes Obes. 2014;2(2):1031. 25506599 PMC4264048

[14] Wang C-P, Lorenzo C, Habib SL, Jo B, Espinoza SE. Differential effects of metformin on age related comorbidities in older men with type 2 diabetes. J Diabetes Compl. 2017;31(4):679–86. 10.1016/j.jdiacomp.2017.01.013 PMC5654524 28190681

[15] Campbell JM, Bellman SM, Stephenson MD, Lisy K. Metformin reduces all-cause mortality and diseases of ageing independent of its effect on diabetes control: a systematic review and meta-analysis. Ageing Res Rev. 2017;40:31–44. 10.1016/j.arr.2017.08.003. 28802803 10.1016/j.arr.2017.08.003

[16] Kulkarni AS, Gubbi S, Barzilai N. Benefits of metformin in attenuating the hallmarks of aging. Cell Metab. 2020;32(1):15–30. 10.1016/j.cmet.2020.04.001. 32333835 10.1016/j.cmet.2020.04.001 PMC7347426

[17] Espinoza SE, Khosla S, Baur JA, de Cabo R, Musi N. Drugs targeting mechanisms of aging to delay age-related disease and promote healthspan: proceedings of a National Institute on Aging Workshop. J Gerontol: Ser A. 2023;78(Supplement_1):53–60. 10.1093/gerona/glad034 PMC10272987 37325957

[18] Mone P, Ciccarelli M, Jankauskas SS, et al. SGLT2 inhibitors and GLP-1 receptor agonists: which is the best anti-frailty drug? Lancet Healthy Longev. 2024;5(9):100632. 10.1016/j.lanhl.2024.08.001 PMC11472278 39284335

[19] Santulli G, Varzideh F, Forzano I, et al. Functional and clinical importance of SGLT2-inhibitors in Frailty: from the kidney to the heart. Hypertension. 2023;80(9):1800–9. 10.1161/hypertensionaha.123.20598. 37403685 10.1161/HYPERTENSIONAHA.123.20598 PMC10529735

[20] Anisimov VN, Berstein LM, Egormin PA, et al. Metformin slows down aging and extends life span of female SHR mice. Cell Cycle (Georgetown, Tex). 2008;7(17):2769–73. 10.4161/cc.7.17.6625. 18728386 10.4161/cc.7.17.6625

[21] Espinoza SE, Musi N, Wang C-P, et al. Rationale and study design of a randomized clinical trial of metformin to prevent frailty in older adults with prediabetes. J Gerontol A Biol Sci Med Sci. 2020;75(1):102–9. 30888034 10.1093/gerona/glz078 PMC7175970

[22] Luchsinger JA, Perez T, Chang H, et al. Metformin in amnestic mild cognitive impairment: results of a pilot randomized placebo controlled clinical trial. J Alzheimers Dis. 2016;51(2):501–14. 10.3233/JAD-150493. 26890736 10.3233/JAD-150493 PMC5079271

[23] Group AI. Study design of ASPirin in Reducing Events in the Elderly (ASPREE): a randomized, controlled trial. Contemp Clin Trials. 2013;36(2):555–64. 24113028 10.1016/j.cct.2013.09.014 PMC3919683

[24] McNeil JJ, Woods RL, Nelson MR, et al. Baseline characteristics of participants in the ASPREE (ASPirin in Reducing Events in the Elderly) Study. J Gerontol Ser A: Biomed Sci Med Sci. 2017;72(11):1586–93. 10.1093/gerona/glw342 PMC5861878 28329340

[25] McNeil JJ, Woods RL, Nelson MR, et al. Effect of aspirin on disability-free survival in the healthy elderly. N Engl J Med. 2018;379(16):1499–508. 10.1056/NEJMoa1800722. 30221596 10.1056/NEJMoa1800722 PMC6426126

[26] Polkinghorne KR, Wolfe R, Jachno KM, et al. Prevalence of chronic kidney disease in the elderly using the ASPirin in Reducing Events in the Elderly study cohort. Nephrology. 2019;24(12):1248–56. 30663195 10.1111/nep.13565 PMC6812602

[27] Ryan J, Woods RL, Britt C, et al. Normative performance of healthy older individuals on the Modified Mini-Mental State (3MS) examination according to ethno-racial group, gender, age, and education level. Clin Neuropsychol. 2019;33(4):779–97. 29976121 10.1080/13854046.2018.1488996 PMC6810645

[28] Zoungas S, Zhou Z, Owen AJ, et al. Daily low-dose aspirin and incident type 2 diabetes in community-dwelling healthy older adults: a post-hoc analysis of efficacy and safety in the ASPREE randomised placebo-controlled trial. Lancet Diabetes Endocrinol. 2024;12(2):98–106. 38142708 10.1016/S2213-8587(23)00327-3 PMC13265035

[29] Espinoza SE, Woods RL, Ekram A, et al. The effect of low-dose aspirin on frailty phenotype and frailty index in community-dwelling older adults in the ASPirin in Reducing Events in the Elderly study. J Gerontol A Biol Sci Med Sci. 2022;77(10):2007–14. 34758073 10.1093/gerona/glab340 PMC9536436

[30] Moritz S, Bartz-Beielstein T. imputeTS: time series missing value imputation in R. R J. 2017;9(1):207.

[31] Ryan J, Espinoza S, Ernst ME, et al. Validation of a deficit-accumulation Frailty Index in the ASPREE study and its predictive capacity for disability-free survival. J Gerontol A Biol Sci Med Sci. 2021.10.1093/gerona/glab225. 10.1093/gerona/glab225 PMC8751791 34338761

[32] Pajewski NM, Williamson JD, Applegate WB, et al. Characterizing frailty status in the systolic blood pressure intervention trial. J Gerontol Ser A: Biomed Sci Med Sci. 2016;71(5):649–55. 10.1093/gerona/glv228 PMC5007741 26755682

[33] Qaseem A, Humphrey LL, Sweet DE, Starkey M, Shekelle P, Clinical Guidelines Committee of the American College of Physicians*. Oral pharmacologic treatment of type 2 diabetes mellitus: a clinical practice guideline from the American College of Physicians. Ann Inter Med. 2012;156(3):218–31. 10.7326/0003-4819-156-3-201202070-00011. (%m22312141). 10.7326/0003-4819-156-3-201202070-00011 22312141

[34] McCullagh P. Regression models for ordinal data. J Roy Stat Soc: Ser B (Methodol). 1980;42(2):109–27.

[35] Volpato S, Bianchi L, Lauretani F, et al. Role of muscle mass and muscle quality in the association between diabetes and gait speed. Diabetes Care. 2012;35(8):1672–9. 22596176 10.2337/dc11-2202 PMC3402248

[36] Park SW, Goodpaster BH, Strotmeyer ES, et al. Accelerated loss of skeletal muscle strength in older adults with type 2 diabetes: the health, aging, and body composition study. Diabetes Care. 2007;30(6):1507–12. 17363749 10.2337/dc06-2537

[37] Kalyani RR, Metter EJ, Egan J, Golden SH, Ferrucci L. Hyperglycemia predicts persistently lower muscle strength with aging. Diabetes Care. 2015;38(1):82–90. 25392294 10.2337/dc14-1166 PMC4274779

[38] Salom Vendrell C, García Tercero E, Moro Hernández JB, Cedeno-Veloz BA. Sarcopenia as a little-recognized comorbidity of type II diabetes mellitus: a review of the diagnosis and treatment. Nutrients. 2023;15(19). 10.3390/nu15194149. 10.3390/nu15194149 PMC10574035 37836433

[39] Wei S, Nguyen TT, Zhang Y, Ryu D, Gariani K. Sarcopenic obesity: epidemiology, pathophysiology, cardiovascular disease, mortality, and management. Front Endocrinol (Lausanne). 2023;14:1185221. 10.3389/fendo.2023.1185221. 37455897 10.3389/fendo.2023.1185221 PMC10344359

[40] Kalyani RR, Varadhan R, Weiss CO, Fried LP, Cappola AR. Frailty status and altered glucose-insulin dynamics. J Gerontol A Biol Sci Med Sci. 2012;67(12):1300–6. 21873592 10.1093/gerona/glr141 PMC3670159

[41] Kuo C-K, Lin L-Y, Yu Y-H, Wu K-H, Kuo H-K. Inverse association between insulin resistance and gait speed in nondiabetic older men: results from the US National Health and Nutrition Examination Survey (NHANES) 1999–2002. BMC Geriatr. 2009;9(1):49. 19922671 10.1186/1471-2318-9-49 PMC2784762

[42] Lee CG, Boyko EJ, Strotmeyer ES, et al. Association between insulin resistance and lean mass loss and fat mass gain in older men without diabetes mellitus. J Am Geriatr Soc. 2011;59(7):1217–24. 21718263 10.1111/j.1532-5415.2011.03472.x PMC3716256

[43] Srikanth V, Sinclair AJ, Hill-Briggs F, Moran C, Biessels GJ. Type 2 diabetes and cognitive dysfunction-towards effective management of both comorbidities. Lancet Diabetes Endocrinol. 2020;8(6):535–45. 10.1016/s2213-8587(20)30118-2. 32445740 10.1016/S2213-8587(20)30118-2

[44] Wang C-P, Hazuda HP. Better glycemic control is associated with maintenance of lower-extremity function over time in Mexican American and European American older adults with diabetes. Diabetes Care. 2011;34(2):268–73. 21216857 10.2337/dc10-1405 PMC3024332

[45] Lee CG, Boyko EJ, Barrett-Connor E, et al. Insulin sensitizers may attenuate lean mass loss in older men with diabetes. Diabetes Care. 2011;34(11):2381–6. 21926282 10.2337/dc11-1032 PMC3198278

[46] Lee CG, Schwartz AV, Yaffe K, et al. Changes in physical performance in older women according to presence and treatment of diabetes mellitus. J Am Geriatr Soc. 2013;61(11):1872–8. 24219188 10.1111/jgs.12502 PMC3827698

[47] Walston J, McBurnie MA, Newman A, et al. Frailty and activation of the inflammation and coagulation systems with and without clinical comorbidities: results from the Cardiovascular Health Study. Arch Intern Med. 2002;162(20):2333–41. 12418947 10.1001/archinte.162.20.2333

[48] Ferrucci L, Fabbri E. Inflammageing: chronic inflammation in ageing, cardiovascular disease, and frailty. Nat Rev Cardiol. 2018;15(9):505. 30065258 10.1038/s41569-018-0064-2 PMC6146930

[49] Mone P, Gambardella J, Pansini A, et al. Cognitive impairment in frail hypertensive elderly patients: role of hyperglycemia. Cells. 2021;10(8). 10.3390/cells10082115. 10.3390/cells10082115 PMC8391431 34440883

[50] Espinoza SE, Jiwani R, Wang J, Wang C-P. Review of interventions for the frailty syndrome and the role of metformin as a potential pharmacologic agent for frailty prevention. Clin Ther. 2019. 10.1016/j.clinthera.2019.01.006 PMC10279480 30851950

[51] Kulkarni AS, Gubbi S, Barzilai N. Benefits of metformin in attenuating the hallmarks of aging. Cell Metab. 2020. 10.1016/j.cmet.2020.04.001 PMC7347426 32333835

[52] Martin-Montalvo A, Mercken EM, Mitchell SJ, et al. Metformin improves healthspan and lifespan in mice. Nat Commun. 2013;4:2192. 23900241 10.1038/ncomms3192 PMC3736576

[53] Wang CP, Lorenzo C, Espinoza SE. Frailty attenuates the impact of metformin on reducing mortality in older adults with type 2 diabetes. J Endocrinol Diabetes Obes. 2014;2(2):1031. 25506599 PMC4264048

[54] Roumie CL, Chipman J, Min JY, et al. Association of treatment with metformin vs sulfonylurea with major adverse cardiovascular events among patients with diabetes and reduced kidney function. JAMA. 2019;322(12):1167–77. 31536102 10.1001/jama.2019.13206 PMC6753652

[55] Santulli G, Visco V, Varzideh F, et al. Prediabetes increases the risk of frailty in prefrail older adults with hypertension: beneficial effects of metformin. Hypertension. 2024;81(7):1637–43. 10.1161/hypertensionaha.124.23087. 38752357 10.1161/HYPERTENSIONAHA.124.23087 PMC11170724

[56] Piskovatska V, Stefanyshyn N, Storey KB, Vaiserman AM, Lushchak O. Metformin as a geroprotector: experimental and clinical evidence. Biogerontology. 2019;20(1):33–48. 30255224 10.1007/s10522-018-9773-5

[57] Espinoza SE, Musi N, Wang C-P, et al. Rationale and study design of a randomized clinical trial of metformin to prevent frailty in older adults with prediabetes. J Gerontol: Ser A. 2019 10.1093/gerona/glz078 PMC7175970 30888034

[58] Strong R, Miller RA, Astle CM, et al. Nordihydroguaiaretic acid and aspirin increase lifespan of genetically heterogeneous male mice. Aging Cell. 2008;7(5):641–50. 18631321 10.1111/j.1474-9726.2008.00414.x PMC2695675

[59] Parish AJ, Swindell WR. Metformin has heterogeneous effects on model organism lifespans and is beneficial when started at an early age in Caenorhabditis elegans: a systematic review and meta-analysis. Aging Cell. 2022;21(12):e13733. 36281624 10.1111/acel.13733 PMC9741508

[60] Holman RR, Paul SK, Bethel MA, Matthews DR, Neil HAW. 10-year follow-up of intensive glucose control in type 2 diabetes. N Engl J Med. 2008;359(15):1577–89. 18784090 10.1056/NEJMoa0806470

[61] Tahmi M, Luchsinger JA. Metformin in the prevention of Alzheimer’s disease and Alzheimer’s disease related dementias. J Prev Alzheimer’s Dis. 2023;10(4):706–17. 37874091 10.14283/jpad.2023.113

[62] Mone P, Martinelli G, Lucariello A, et al. Extended-release metformin improves cognitive impairment in frail older women with hypertension and diabetes: preliminary results from the LEOPARDESS Study. Cardiovasc Diabetol. 2023;22(1):94. 10.1186/s12933-023-01817-4. 37085892 10.1186/s12933-023-01817-4 PMC10122301

[63] Mone P, Varzideh F, Jankauskas SS, et al. SGLT2 inhibition via empagliflozin improves endothelial function and reduces mitochondrial oxidative stress: insights from frail hypertensive and diabetic patients. Hypertension. 2022;79(8):1633–43. 10.1161/hypertensionaha.122.19586. 35703100 10.1161/HYPERTENSIONAHA.122.19586 PMC9642044

[64] Cortes TM, Vasquez L, Serra MC, et al. Effect of semaglutide on physical function, body composition, and biomarkers of aging in older adults with overweight and insulin resistance: protocol for an open-labeled randomized controlled trial. JMIR Res Protoc. 2024;13(1):e62667. 39269759 10.2196/62667 PMC11437224

[65] Cheng M, He M, Ning L, et al. The impact of frailty on clinical outcomes among older adults with diabetes: a systematic review and meta-analysis. Medicine. 2024;103(26):e38621. 10.1097/md.0000000000038621. 38941383 10.1097/MD.0000000000038621 PMC11466167

[66] Liu Y, Zhang L, Li X, et al. Prevalence and risk factors of frailty in older adults with diabetes: a systematic review and meta-analysis. PLoS One. 2024;19(10):e0309837. 39480799 10.1371/journal.pone.0309837 PMC11527323

[67] Strain WD, Down S, Brown P, Puttanna A, Sinclair A. Diabetes and frailty: an expert consensus statement on the management of older adults with type 2 diabetes. Diabetes Therapy. 2021;12(5):1227–47. 33830409 10.1007/s13300-021-01035-9 PMC8099963

[68] Care D. 13. Older adults: standards of medical care in diabetes—2022. Diabetes Care. 2022;45:S195. 34964847 10.2337/dc22-S013 PMC8935395

