# Supplementary material for: Frailty incidence by diabetes treatment regimens in older adults with diabetes mellitus in the ASPirin in Reducing Events in the Elderly Study
Source: GeroScience. 2025 Mar 17;47(3):5169–83. doi: 10.1007/s11357-025-01598-6 (PMC12181461; doi:10.1007/s11357-025-01598-6)
Supplement: Supplementary file 1 — Supplementary file1 (DOCX 25.4 KB) [file 11357_2025_1598_MOESM1_ESM.docx]

| **Supplemental Table 1. Diabetes medications use by other diabetes medication only and combined metformin and other diabetes medication only groups** | | |
| --- | --- | --- |
| **Medication** | **Other diabetic medications only**  **(N = 200)** | **Metformin and other diabetic medications**  **(N = 420)** |
| **Sulfonylureas** |  |  |
| Gliclazide | 63 (31.5%) | 162 (38.57%) |
| Glipizide | 21 (10.5%) | 31 (7.38%) |
| Glibenclamide | 15 (7.5%) | 40 (9.52%) |
| Glimepiride | 13 (6.5%) | 41 (9.76%) |
| **Alpha-glucosidase inhibitors** |  |  |
| Acarbose | 1 (0.5%) | 3 (0.71%) |
| **Thiazolidinediones** |  |  |
| Pioglitazone | 15 (7.5%) | 32 (7.62%) |
| Rosiglitazone | 1 (0.5%) | 6 (1.43%) |
| **DPP-4 inhibitors**^a^ |  |  |
| Sitagliptin | 25 (12.5%) | 60 (14.29%) |
| Vildagliptin | 0 (0%) | 14 (3.33%) |
| Saxagliptin | 5 (2.5%) | 10 (2.38%) |
| Linagliptin | 3 (1.5%) | 6 (1.43%) |
| **GLP-1 agonists**^b^ |  |  |
| Exenatide | 2 (1%) | 10 (2.38%) |
| Liraglutide | 0 (0%) | 1 (0.24%) |
| **SGLT-2 inhibitor**^c^ |  |  |
| Canagliflozin | 0 (0%) | 2 (0.48%) |
| **Meglitinide** |  |  |
| Repaglinide | 1 (0.5%) | 2 (0.48%) |
| **Insulin** | 74 (37%) | 83 (19.76%) |

^a^Dipeptidyl peptidase-4 inhibitor

^b^Glucagon-like peptide-1 agonist

^c^Sodium-glucose Cotransporter-2 inhibitor

| **Supplemental Table 2. Time to frailty event (imputed) by diabetes medication group** | | | | |
| --- | --- | --- | --- | --- |
| **Endpoint** | **Metformin only** | **No diabetes medications** | **Metformin and other diabetes medications** | **Other diabetes medications only** |
| Number of events and rates per 1000 person-years | | | | |
| Fried frailty^a^ | 75 (51.99) | 111 (47.08) | 65 (58.23) | 42 (90.42) |
| FI frailty^b^ | 117 (84.58) | 178 (74.38) | 91 (86.95) | 44 (103.71) |
| Cox proportional-hazards regression models | | | | |
| Reference group: Other diabetes medications only | | | | |
| Fried frailty^ac^ | 0.63  (0.41, 0.97) | 0.69  (0.46, 1.05) | 0.79  (0.51, 1.21) | Ref |
| FI frailty^bc^ | 0.72  (0.49, 1.05) | 0.68  (0.47, 0.99) | 0.70  (0.48, 1.03) | Ref |
| Fried frailty (adjusted for baseline Fried score)^ac^ | 0.79  (0.52, 1.21) | 0.94  (0.61, 1.44) | 0.94  (0.61, 1.45) | Ref |
| FI frailty (adjusted for baseline FI score)^bc^ | 0.87  (0.60, 1.28) | 0.75  (0.52, 1.09) | 0.87  (0.59, 1.29) | Ref |
| Reference group: No diabetes medications | | | | |
| Fried frailty^ac^ | 0.91  (0.65, 1.26) | Ref | 1.13  (0.79, 1.63) | 1.44  (0.95, 2.19) |
| FI frailty^bc^ | 1.05  (0.81, 1.36) | Ref | 1.03  (0.76, 1.39) | 1.46  (1.01, 2.12) |
| Fried frailty (adjusted for baseline Fried score)^ac^ | 0.84  (0.60, 1.17) | Ref | 1.00  (0.69, 1.44) | 1.06  (0.70-1.63) |
| FI frailty (adjusted for baseline FI score)^bc^ | 1.16  (0.89, 1.51) | Ref | 1.16  (0.86, 1.57) | 1.33  (0.92, 1.93) |
| Reference group: Metformin and other diabetes medications | | | | |
| Fried frailty^ac^ | 0.80  (0.56, 1.14) | 0.88  (0.61, 1.27) | Ref | 1.27  (0.83, 1.95) |
| FI frailty^bc^ | 1.02  (0.76, 1.37) | 0.97  (0.72, 1.31) | Ref | 1.42  (0.97, 2.10) |
| Fried frailty (adjusted for baseline Fried score)^ac^ | 0.84  (0.59, 1.21) | 1.00  (0.69, 1.44) | Ref | 1.06  (0.69, 1.65) |
| FI frailty (adjusted for baseline FI score)^bc^ | 1.00  (0.75, 1.34) | 0.86  (0.64, 1.17) | Ref | 1.15  (0.78, 1.70) |
| Reference group: Metformin only | | | | |
| Fried frailty^ac^ | Ref | 1.10  (0.79, 1.53) | 1.25  (0.88, 1.77) | 1.58  (1.04, 2.42) |
| FI frailty^bc^ | Ref | 0.95  (0.74, 1.24) | 0.98  (0.73, 1.31) | 1.40  (0.95, 2.04) |
| Fried frailty (adjusting for baseline Fried score)^ac^ | Ref | 1.19  (0.85, 1.65) | 1.19  (0.83, 1.69) | 1.26  (0.82, 1.94) |
| FI frailty (adjusting for baseline FI score)^bc^ | Ref | 0.87  (0.67, 1.13) | 1.02  (0.77, 1.37) | 1.11  (0.76, 1.61) |

^a^Fried frail endpoint is defined as the presence of three or more Fried frailty phenotype criteria. Participants who were frail at baseline or had missing frailty data at baseline were excluded.

^b^FI frail endpoint is defined as deficit accumulation FI score of greater than 0.21. Participants who were frail at baseline or had frailty data at baseline were excluded.

^c^Adjusted for age, sex, education, ethnicity, alcohol use, antihypertensive use, SBP, DBP, triglycerides, HDL, LDL, statins use, hemoglobin, eGFR, polypharmacy (5+ medications), 3MS, fasting blood glucose and BMI.

| **Supplemental Table 3. Time to frailty event (non-imputed) by diabetes medication group** | | | | |
| --- | --- | --- | --- | --- |
| **Endpoint** | **Metformin only** | **No diabetes medications** | **Metformin and other diabetes medications** | **Other diabetes medications only** |
| Number of events and rates per 1000 person-years | | | | |
| Fried frailty^a^ | 44 (29.6) | 64 (26.82) | 46 (40.66) | 26 (54.9) |
| FI frailty^b^ | 114 (81.3) | 177 (73.65) | 88 (83.83) | 41 (94.86) |
| Cox proportional-hazards regression models | | | | |
| Reference group: Other diabetes medications only | | | | |
| Fried frailty^ac^ | 0.67  (0.38, 1.16) | 0.65  (0.37, 1.13) | 1.05  (0.61, 1.81) | Ref |
| FI frailty^bc^ | 0.76  (0.52, 1.13) | 0.76  (0.52, 1.11) | 0.75  (0.50, 1.12) | Ref |
| Fried frailty (adjusted for baseline Fried score)^ac^ | 0.82  (0.47, 1.43) | 0.83  (0.47, 1.45) | 1.23  (0.71, 2.13) | Ref |
| FI frailty (adjusted for baseline FI score)^bc^ | 0.94  (0.63, 1.39) | 0.85  (0.58, 1.24) | 0.95  (0.64, 1.43) | Ref |
| Reference group: No diabetes medications | | | | |
| Fried frailty^ac^ | 1.03  (0.66, 1.59) | Ref | 1.62  (1.03, 2.54) | 1.54  (0.88, 2.69) |
| FI frailty^bc^ | 1.01  (0.78, 1.31) | Ref | 1.00  (0.73, 1.35) | 1.32  (0.90, 1.94) |
| Fried frailty (adjusted for baseline Fried score)^ac^ | 0.99  (0.64, 1.54) | Ref | 1.49  (0.94, 2.36) | 1.21  (0.69, 2.13) |
| FI frailty (adjusted for baseline FI score)^bc^ | 1.11  (0.85, 1.45) | Ref | 1.13  (0.83, 1.53) | 1.18  (0.81, 1.73) |
| Reference group: Metformin and other diabetes medications | | | | |
| Fried frailty^ac^ | 0.64  (0.41, 0.99) | 0.62  (0.39, 0.97) | Ref | 0.95  (0.55, 1.65) |
| FI frailty^bc^ | 1.01  (0.75, 1.37) | 1.00  (0.74, 1.36) | Ref | 1.33  (0.89, 1.98) |
| Fried frailty (adjusted for baseline Fried score)^ac^ | 0.67  (0.43, 1.04) | 0.67  (0.42, 1.06) | Ref | 0.81  (0.47, 1.41) |
| FI frailty (adjusted for baseline FI score)^bc^ | 0.99  (0.73, 1.32) | 0.89  (0.65, 1.20) | Ref | 1.05  (0.70, 1.57) |
| Reference group: Metformin only | | | | |
| Fried frailty^ac^ | Ref | 0.97  (0.63, 1.51) | 1.57  (1.01, 2.44) | 1.50  (0.86, 2.62) |
| FI frailty^bc^ | Ref | 0.99  (0.76, 1.29) | 0.99  (0.73, 1.33) | 1.31  (0.89, 1.94) |
| Fried frailty (adjusted for baseline Fried score)^ac^ | Ref | 1.01  (0.65, 1.57) | 1.50  (0.96, 2.35) | 1.22  (0.70, 2.13) |
| FI frailty (adjusted for baseline FI score)^bc^ | Ref | 0.90  (0.69, 1.17) | 1.01  (0.76, 1.36) | 1.06  (0.72, 1.57) |

^a^Fried frail endpoint is defined as the presence of three or more Fried frailty phenotype criteria. Participants who were frail at baseline or had missing frailty data at baseline were excluded.

^b^FI frail endpoint is defined as deficit accumulation FI score of greater than 0.21. Participants who were frail at baseline or had frailty data at baseline were excluded.

^c^Adjusted for age, sex, education, ethnicity, alcohol use, antihypertensive use, SBP, DBP, triglycerides, HDL, LDL, statins use, hemoglobin, eGFR, polypharmacy (5+ medications), 3MS, fasting blood glucose and BMI.
