# Supplementary material for: Frailty incidence by diabetes treatment regimens in older adults with diabetes mellitus in the ASPirin in Reducing Events in the Elderly Study
Source: GeroScience. 2025 Mar 17;47(3):5169–83. doi: 10.1007/s11357-025-01598-6 (PMC12181461; doi:10.1007/s11357-025-01598-6)

**Supplemental Figure 1**. Flow diagram of the ASPREE participants with diabetes. The numbers of participants with self-report diabetes, elevated blood glucose, and anti-diabetic medication use are not mutually exclusive. Note that those with who are frail at baseline are included in the figure above, as they were included in the mixed effects ordinal logistic regression models, though not included in the Cox proportional hazards regression models.


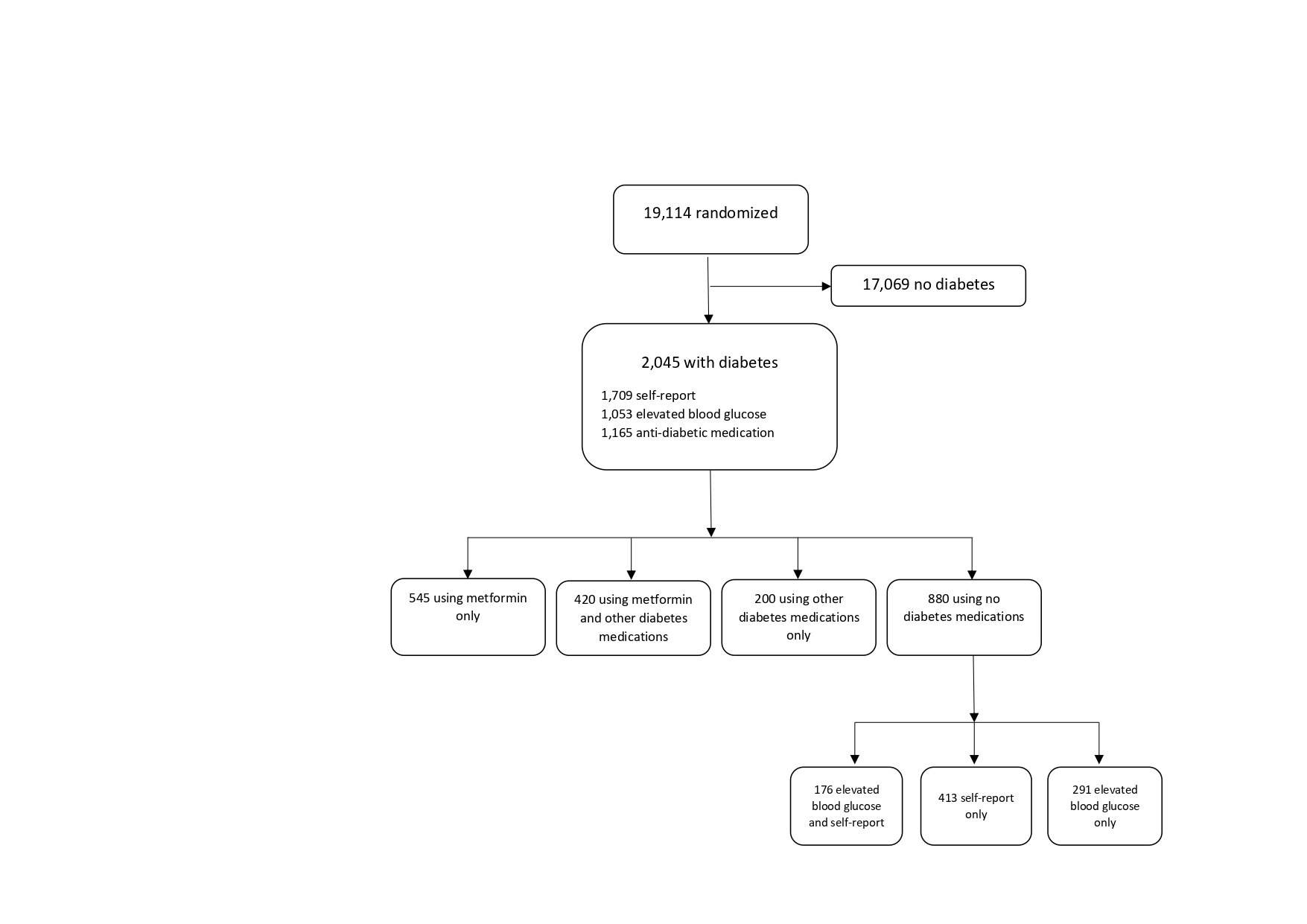

Supplement: Supplementary file 2 — Supplementary file2 (DOCX 128 KB) [file 11357_2025_1598_MOESM2_ESM.docx]
